# Water table management and fertilizer application impacts on CO_2_, N_2_O and CH_4_ fluxes in a corn agro-ecosystem

**DOI:** 10.1038/s41598-019-39046-z

**Published:** 2019-02-25

**Authors:** Cynthia M. Crézé, Chandra A. Madramootoo

**Affiliations:** 1 Department of Bioresource Engineering, Macdonald Campus, McGill University, 21,111 Lakeshore Road, Sainte-Anne-de-Bellevue, Québec H9X 3V9 Canada; 2 Present Address: Department of Plant Sciences, University of California at Davis, One Shields Avenue, Davis, CA 95616 USA

## Abstract

Water table management with controlled drainage and subsurface-irrigation (SI) has been identified as a Beneficial Management Practice (BMP) to reduce nitrate leaching in drainage water. It has also been shown to increase crop yields during dry periods of the growing season, by providing water to the crop root zone, via upward flux or capillary rise. However, by retaining nitrates in anoxic conditions within the soil profile, SI could potentially increase greenhouse gas (GHG) fluxes, particularly N_2_O through denitrification. This process may be further exacerbated by high precipitation and mineral N-fertilizer applications very early in the growing season. In order to investigate the effects of water table management (WTM) with nitrogen fertilization on GHG fluxes from corn (*Zea mays*) agro-ecosystems, we conducted a research study on a commercial farm in south-western Quebec, Canada. Water table management treatments were: free drainage (FD) and controlled drainage with subsurface-irrigation. GHG samples were taken using field-deployed, vented non-steady state gas chambers to quantify soil CO_2_, N_2_O and CH_4_ fluxes weekly. Our results indicate that fertilizer application timing coinciding with intense (≥24 mm) precipitation events and high temperatures (>25 °C) triggered pulses of N_2_O fluxes, accounting for up to 60% of cumulative N_2_O fluxes. Our results also suggest that splitting bulk fertilizer applications may be an effective mitigation strategy, reducing N_2_O fluxes by 50% in our study. In both seasons, pulse GHG fluxes mostly occurred in the early vegetative stages of the corn, prior to activation of the subsurface-irrigation. Our results suggest that proper timing of WTM mindful of seasonal climatic conditions has the potential to reduce GHG emissions.

## Introduction

Subsurface pipe drainage, or tile drainage, is essential for crop production in Eastern Canada. Under conventional tile drainage or free drainage, a field can be freely drained of excess water facilitating spring field operations. FD also facilitates the removal of excess precipitation during the growing season. With environmental concerns regarding excessive nutrients (N, P) being leached to surface water bodies via tile drains, water table management systems, such as controlled drainage and subsurface-irrigation, are being suggested. With SI, drain discharge is restricted, thereby creating an elevated water table in the field. In addition, supplemental subsurface-irrigation water can be pumped through the drainage system to maintain the water table depth (WTD) at a target level. Although SI systems have the ability to provide supplemental water in periods of high seasonal evapotranspiration, SI systems are installed by crop growers primarily to improve field drainage in the spring and to retain nutrients in the soil profile. As such, this system is very different from other irrigation systems such as drip, sprinkler, center-pivot and furrow, where water is applied by surface methods.

Our study is a part of a long-term assessment of SI technology conducted at St-Emmanuel, southern Quebec since 1993. The research results have shown that SI reduced nitrate losses to the environment due to three principal mechanisms: (i) nitrate retention in the soil matrix, available for plant uptake later in the growing season, (ii) slower nitrification processes due to greater soil moisture from an imposed, elevated, controlled water table, and (iii) enhanced denitrification, due to higher dissolved organic carbon, which occurs prior to nitrates being leached to the groundwater^1–11^. Hence, SI systems have been identified as a BMP to reduce nitrate leaching in drainage water, and is well-documented in previous literature^12–14^.

Our GHG study builds upon previous work at the St-Emmanuel site, which focused on N leaching and corn N recovery, and therefore enlarges our collective understanding of N dynamics in subsurface-irrigated maize fields. As soil substrates are retained within the rhizosphere, SI improves the availability of nutrients for crop uptake. Field-level evaluations of this system have indicated higher corn yields in SI compared to FD^15–19^. This study questions whether SI could in fact stimulate GHG efflux by retaining key components of denitrification, particularly nitrates and water above the tile drains. Previous results from Elmi *et al*. (ref.^7^) using the intact soil core method found greater denitrification rates under SI compared to FD systems. However, results of the study suggested that due to the depth of the anoxic zone in SI plots, the N_2_O produced may further be reduced to N_2_, before it diffuses to the atmosphere. As of now, *in-situ* field measurements have not been conducted on this site to verify this process. Furthermore, studies reporting the effect of fertilizer on GHG efflux in subsurface-irrigated fields of Eastern Canada remain scarce.

Here we assessed how a combination of factors, particularly mineral N-fertilizer application, water table management and precipitation events affect CO_2_, N_2_O and CH_4_ soil-atmosphere exchange. An important feature of our study is that it was done on a large commercial field scale, rather than on small experimental plots, so that farm operator practices could be taken into account. The main objectives of our study were to: (i) compare fluxes of soil CO_2_, N_2_O and CH_4_ from commercial corn fields under conventional tile drainage and water table management in the form of subsurface-irrigation, and (ii) study the effects of fertilizer applications on GHG fluxes. We hypothesized that although subsurface-irrigation has been demonstrated to increase denitrification rates within the soil profile, GHG efflux from the soil surface would not be increased. Furthermore, splitting fertilizer applications would have a greater effect compared to WTM on decreasing gas fluxes by providing a slower input of substrates to the soil.

## Results

### Meteorological Data

**Figure 1:**
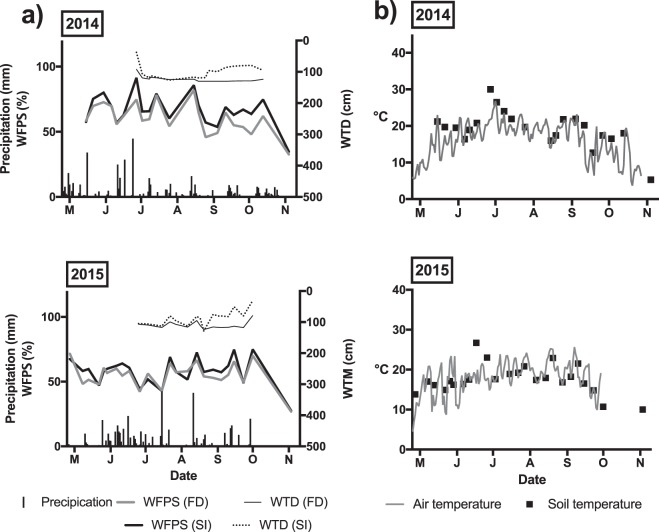
(**a**) Precipitation, water filled pore space (WFPS), and water table depth (WTD) for free drainage (FD) and subsurface-irrigation (SI) plots for 2014 and 2015. The detection limit of the WTD was of 130 cm. (**b**) Air and soil temperature (°C) for 2014 and 2015.

The crop growing seasons of both 2014 and 2015 were drier and slightly warmer than the 40-year regional average. In 2014 and 2015 respectively, seasonal rainfall amounts from May to the end of September were 465 and 494 mm, which were 15% and 10% below the 40-year average for the experimental site. Mean seasonal air temperatures were 17 °C in 2014 and 18 °C in 2015. These were 2% and 8%, respectively, higher than the 40-year average. The month of June received the most rainfall for both growing seasons, accounting for 28% and 27% of the respective seasonal total rainfalls. June 2014 was characterized by three separate events of daily precipitations of more than 20 mm followed by days with no more than 15 mm on any one day. June 10 (25 mm) was followed by 5 days with no more than 15 mm, with 6 mm and 15 mm on June 11 and June 12 respectively, followed by 3 rainless days. June 16 (29 mm) was followed by 6 days with less than 2 mm of rain daily. June 23 (45 mm) was followed by 8 days with no more than 2 mm of rain daily. In contrast, June 2015 had a similar monthly rainfall amount to June 2014, but distributed in equal and more frequent events. Consequentially, in 2015, soil water-filled pore space (WFPS) in the top 10 cm of the soil was uniform during the season and did not show the same extent of fluctuations as 2014, which fluctuated by up to 15% (Fig. 1). Warmest monthly temperatures were in July and were 14% and 11% greater than the seasonal average temperatures in 2014 and 2015, respectively (Fig. 1). Soil temperatures at a 10 cm depth ranged from 5–30 °C in 2014 and from 11–27 °C in 2015. The highest weekly average soil temperatures occurred on June 27, 2014 (30 °C) and, on June 17, 2015 (27 °C).

### Precipitation and water table management effects on soil water-filled pore space

The average recorded water table depth in SI plots was 83 cm (SD ± 11) in 2014 and 81 cm (SD ± 13) in 2015. Based on weekly measurements, subsurface-irrigation increased topsoil water-filled pore space by 4% on average in 2014 and by 1% in 2015. In contrast, daily precipitation events of more than 30 mm created increases in WFPS of up to 30% in the top 10 cm of the soil, which were sometimes followed by decreases in WFPS of ≥20% due to evapotranspiration and percolation (Fig. 1). Even at times of uniform water table levels, large fluctuations in soil WFPS were observed, as a result of high precipitation amounts (Fig. 1**)**. Compared to subsurface-irrigation treatments, precipitation had a stronger effect on the water content of the top 10 cm of the soil, the depth at which the GHG chamber frames were inserted.

### Greenhouse gas fluxes

#### CO_2_ flux

**Figure 2:**
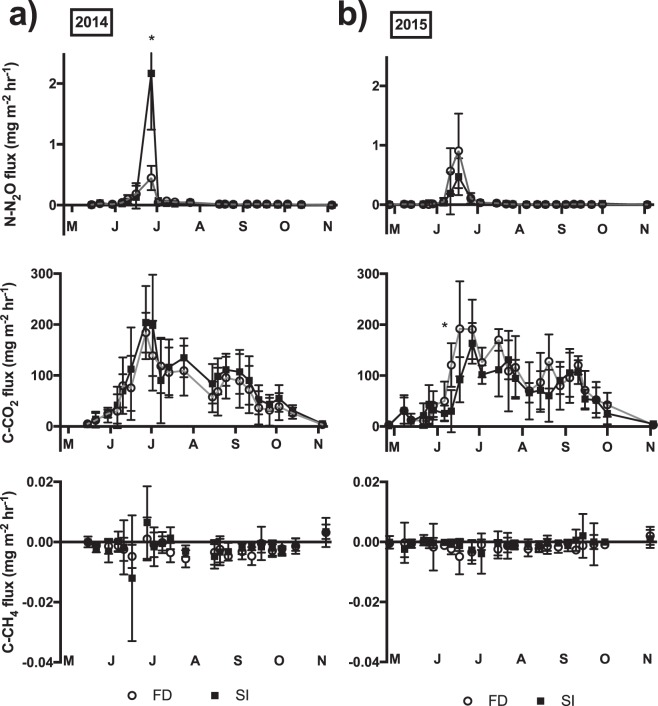
Mean GHG fluxes (mg m^−2^ hr^−1^) and standard deviation under free drainage (FD) and subsurface-irrigation (SI) treatments in 2014 (**a**) and 2015 (**b**). An SD bar was clipped at the axis limit for N_2_O in 2014. Asterisks indicate statistically significant differences between water treatments (P < 0.05).

In 2014, daily CO_2_ fluxes ranged from 0.23 to 220 mg C-CO_2_/m^2^/hr for FD and from zero to 330 mg C-CO_2_/m^2^/hr for SI plots, as compared to a baseline value of 1.16 (SD ±0.71) mg C-CO_2_/m^2^/hr taken on November 7, 2013 at the experimental site (Fig. 2). Carbon dioxide flux equivalents were as follows: 0.06 to 52.9 kg/ha/day for FD, zero to 79.2 kg/ha/day for SI and 0.28 kg/ha/day as a baseline. Carbon dioxide fluxes from subsurface-irrigated plots were statistically significantly higher than in freely drained plots (P < 0.05), with the average seasonal flux of SI plots being 21% greater than that of FD plots (Table 1). However, our results indicated that CO_2_ fluxes were not significantly correlated to seasonal changes in soil WFPS (*r* = 0.02, P = 0.89). Flux values from SI plots were on average 29% and 13% greater than from FD plots for the months of June and July, respectively. Two elevated CO_2_ fluxes (293 and 330 mg C-CO_2_/m^2^/hr) were captured from individual chambers in SI plots on June 27 and July 2, 2014. The substantial increases of CO_2_ observed during June 2014 were observed 20 days following a bulk fertilizer application of 160 kg N/ha applied on June 7 (Fig. 2).

**Table 1:** Mean daily N_2_O, CO_2_ and CH_4_ fluxes by treatment for 2014 and 2015.

			Average mean daily flux		
			N_2_O (S.D.)	CO_2_ (S.D.)	CH_4_ (S.D.)
			mg N-N_2_O.m^−2^.hr^−1^	mg C-CO_2_.m^−2^.hr^−1^	mg C-CH_4_.m^−2^.hr^−1^
2014	Water treatment	FD	0.055 (0.039)	66.9 (8.9)	−0.002 (0.001)
		SI	0.131*** (0.069)	80.2**(8.9)	−0.001 (0.001)
2015	Water treatment	FD	0.074** (0.053)	80.5***(6.0)	−0.001 (0.000)
		SI	0.042 (0.039)	63.6 (6.0)	−0.001** (0.000)
	Fertilizer treatment	Bulk	0.079**(0.049)	72.4 (6.0)	−0.001 (0.000)
		Split	0.037 (0.037)	71.8 (6.0)	−0.001 (0.000)

Significance levels are indicated for the treatments that had significantly higher fluxes. **p<0.05–0.01; ***p<0.01.

In 2015, four treatment combinations were studied: free drainage with one bulk application of 200 kg N/ha (F1), FD with two applications of 100 kg N/ha spaced one week apart (F2), SI with F1 and SI with F2. Subsurface-irrigation treatments were found to have a statistically significant effect (P<0.01) on CO_2_ fluxes, with the average seasonal flux of SI plots 21% lower than that of FD plots. Mean daily CO_2_ fluxes in 2015 ranged from 0.47 to 275 mg C-CO_2_/m^2^/hr in FD plots and from 0.29 to 211 mg C-CO_2_/m^2^/hr in SI plots. Carbon dioxide flux equivalents were as follows: 0.11 to 65.9 kg/ha/day for FD and 0.07 to 50.7 kg/ha/day for SI. Flux values from SI plots were on average 48% and 12% lower than from FD plots for the months of June and July, respectively. Elevated CO_2_ fluxes (269 and 275 mg C-CO_2_/m^2^/hr) were obtained from individual chambers in FD plots on June 17 and June 26, 2015. The significant effect of sampling days on CO_2_ fluxes (P < 0.01) can partially be attributed to seasonal variations in soil temperature with which CO_2_ was positively correlated (*r* = 0.74, P < 0.01), as indicated in Supplementary Table S2. Furthermore, these peak CO_2_ fluxes occurred within 20 days of bulk fertilizer applications on May 29 for F1 plots, and on May 29 and June 3 for F2 plots. However, based on ANOVA results, fertilizer treatments did not have a statistically significant effect (P = 0.90) on CO_2_ fluxes in 2015, as indicated in Supplementary Table S1.

#### N_2_O flux

In 2014, daily N_2_O fluxes ranged from −0.012 to 0.694 mg N-N_2_O/m^2^/hr in FD plots and from −0.001 to 3.89 mg N-N_2_O/m^2^/hr in SI plots (Fig. 2), compared to baseline values which indicated N_2_O fluxes of 0.001 (SD ±0.001) mg N-N_2_O/m^2^/hr. Nitrous oxide flux equivalents were as follows: −2.95 to 167 g/ha/day for FD plots, −0.139 to 935 g/ha/day for SI plots and 0.168 g/ha/day as the baseline. Subsurface-irrigation effects on N_2_O fluxes were found to be statistically significant (P < 0.01). Overall, average seasonal fluxes of SI plots were 2.3 times that of FD plots. Peak N_2_O fluxes in SI and FD plots occurred when soil WFPS were 91% and 75%, respectively, which were among the highest values recorded in this study. High N_2_O fluxes (1.75, 2.04 and 3.89 mg N-N_2_O/m^2^/hr) were recorded from three individual chambers in SI plots on June 27, 2014. These peak values were respectively 7, 5 and 4 times higher than the mean flux from FD plots on that same day (0.446 mg N-N_2_O/m^2^/hr). However, our results indicated that the positive correlation between WFPS and N_2_O fluxes was not significant (*r* = 0.28, P  = 0.06). Peak N_2_O fluxes were recorded 20 days following a bulk application of 160 kg N/ha on June 7, and 4 days after an intense precipitation event of 45 mm on June 23. Peak N_2_O fluxes also coincided with the highest seasonal soil temperatures (Fig. 1). N_2_O fluxes and soil temperature (°C) were positively correlated (*r* = 0.52, P < 0.01) with peak fluxes occurring at 30 °C.

In 2015, both subsurface-irrigation and fertilizer effects on N_2_O fluxes were statistically significant (P < 0.05, P < 0.05, respectively). Of fertilizer treatments, F1 had mean N_2_O fluxes of 0.079 mg N-N_2_O/m^2^/hr, more than twice that of F2 (0.037 mg N-N_2_O/m^2^/hr). Mean daily N_2_O fluxes from F1 ranged from 0.001 mg N-N_2_O/m^2^/hr to peak fluxes reaching 0.919 mg N-N_2_O/m^2^/hr. Mean daily N_2_O fluxes in FD ranged from 0.001 to 0.907 mg N-N_2_O/m^2^/hr compared to ranges of zero to 0.470 mg N-N_2_O/m^2^/hr in SI plots. Nitrous oxide flux equivalents were 0.240 to 218 g/ha/day for FD and zero to 113 g/ha/day for SI. In contrast with 2014, FD had significantly greater N_2_O fluxes (P < 0.05) in 2015 with a mean value of 0.074 mg N-N_2_O/m^2^/hr compared to 0.042 mg N-N_2_O/m^2^/hr in SI plots.

Although the interaction between the two fertilizer and subsurface-irrigation treatments was not significant in 2015 (P > 0.05), sampling day had an important impact on both fertilizer and subsurface-irrigation effects (P < 0.01) suggesting a potential combined influence of seasonal soil temperature and water content on N_2_O fluxes. N_2_O fluxes were significantly greater relative to other treatments only for the FDF1 plots on June 11, 2015 (P < 0.01). On June 17, 2015, SI, FD, F1 and F2 plots all had significantly greater N_2_O fluxes compared to fluxes taken on other sampling days within the same treatment (P < 0.01). Significantly positive correlations were found between N_2_O fluxes and soil temperature in this study (*r* = 0.52, P < 0.01), whereas the positive correlation between soil WFPS and N_2_O fluxes was not significant (*r* = 0.28, P = 0.06). The timing of seasonal N_2_O peaks with respect to field operations was important to consider. Peak fluxes on June 11 and June 17 occurred within 20 days of bulk fertilizer applications on May 29 for F1 plots, and on May 29 and June 3 for F2 plots. The elevated N_2_O flux on June 11, 2015 was recorded after four rainfall days accounting for a total of 42 mm of precipitation. Furthermore, an ‘extreme’ precipitation event of 24 mm occurred on June 16, one day preceding the peak flux recorded on June 17, 2015. This seems to agree with the coupled fertilizer-precipitation effect observed in 2014.

#### CH_4_ flux

In 2014, methane flux means were of −0.002 mg C-CH_4_/m^2^/hr in FD plots and −0.001 mg C-CH_4_/m^2^/hr in SI plots, with a baseline value of zero (SD ±0.001) mg C-CH_4_/m^2^/hr. Methane flux equivalents were as follows: −0.48 g/ha/day for FD, −0.336 g/ha/day for SI and −0.024 g/ha/day as a baseline. The field behaved as a methane sink. Whereas chamber values in FD plots ranged from −0.016 to 0.012 mg C-CH_4_/m^2^/hr, SI plots had two extreme chamber values of both methane consumption and methane production (−0.017 and +0.017 mg C-CH_4_/m^2^/hr). However, overall, the effect of SI on CH_4_ fluxes was not found to be statistically significant (P >0.05). June 16, 2014 had significantly greater methane consumption compared to nine of the 20 other sampling days (P < 0.01). Peak fluxes of CH_4_ coincided with peak NO_2_ and CO_2_ fluxes, recorded 20 days following the bulk fertilizer application. Following seasonal peak methane consumption, peak methane production was recorded 11 days later on June 27 (daily mean of 0.004 mg C-CH_4_/m^2^/hr). These extreme fluxes may have been attributed to seasonally high soil temperatures with 21 °C on June 16, and 30 °C on June 27. Prior to peak methane consumption on June 16, 25 mm of precipitation fell on June 10 followed by 15 mm on June 12 with a total rainfall amount of 46 mm in the week preceding gas sampling. Similarly, peak methane production on June 27 followed 4 days after an intense precipitation event of 45 mm on June 23.

In 2015, subsurface-irrigation was found to have a statistically significant effect on CH_4_ fluxes (P < 0.05). Mean methane fluxes in SI plots were significantly greater than in FD plots (P < 0.05). Methane equivalents were as follows: −0.144 g/ha/day for SI, and −0.336 g/ha/day for FD. Fertilizer treatments did not have statistically significant effects (P = 0.56) on CH_4_ fluxes. However, F1 plots had a lower mean daily flux than F2 plots. The field acted as a methane sink on 21 of the 24 sampling days, with the exception of May 22, September 15 and November 4, at which times, soil temperatures were at seasonal lows. The negative correlation of CH_4_ fluxes with soil temperature (*r* = −0.24, P = 0.12) and with WFPS (*r* = −0.03, P = 0.82) were not found to be significant. The greatest methane consumption occurred on June 17 and June 26, the same days as peak CO_2_ and N_2_O fluxes. For both seasons, peak fluxes of CH_4_ coincided with peak NO_2_ and CO_2_ fluxes.

## Discussion

Our results indicate that fertilizer application timing coupled with precipitation events have stronger effects on N_2_O fluxes than subsurface-irrigation. Single precipitation events of ≥24 mm within three weeks of bulk fertilizer applications led to N_2_O production rates that were 100 times greater in 2014, than the maximum mean value measured from subsurface-irrigated plots, and 45 times greater in 2015. Subsurface-irrigation increased topsoil WFPS by 4% on average in 2014 and by 1% in 2015, whereas daily precipitation events of more than 30 mm led to increases in WFPS of up to 30%, which could be followed by decreases in WFPS of ≥20% (Fig. 1). Literature suggests that the flushing of soil by heavy precipitation may release carbon bound to soil aggregates^20^, resulting in an increase in organic C under wet-dry cycles in the soil, as demonstrated in both field and laboratory experiments^21–24^. Considering the relatively low organic matter content (3.5%) of the study site and the lack of organic inputs (no manure nor compost application), carbon could be expected to be a limiting element. Released carbon following precipitation can in turn accelerate microbial activity in the rhizosphere, which further contributes to elevated CO_2_ fluxes. The peak CO_2_ fluxes observed in our study also occurred during the period prior to corn tasseling, which corresponds to the vegetative stage during which most of the root development occurs. Roots can contribute directly to CO_2_ efflux through respiration but also indirectly by supplying C compounds to the soil through exudation. Rochette *et al*. (ref.^25^) reported that roots and associated microorganisms could account for up to 45% of the total respiration in a corn field, most of which occurs in mid-summer. Later in the season, pulse CO_2_ and N_2_O pulses may be restricted due to crop N uptake resulting in diminishing soil available nitrates, as previously suggested by Elmi *et al*. (ref.^26^). Temperature is a driving factor of these mechanisms and was a key regulator of CO_2_ efflux in our study. Soil temperature had a stronger positive correlation to CO_2_ than WFPS and accounted for >50% of the variation in fluxes. Peak CO_2_ fluxes coincided with the highest seasonal temperatures (>25 °C) occurring in mid-summer of both seasons. Temperature control on soil respiration has been well-documented, with the optimum temperature for soil respiration being at 30 °C^27,28^. However, in this study, highest mean CO_2_ fluxes following coupled precipitation-fertilizer events (192 mg/m^2^/hr in FD and 204 mg/m^2^/hr in SI) were similar to those obtained by Edwards *et al*. (ref.^29^), Kallenback *et al*. (ref.^21^) and Burger *et al*. (ref.^30^), reporting peak values of 200 mg/m^2^/hr. Fluxes of CO_2_ may have been limited by the remaining labile carbon pool obstructing soil respiration processes in our study.

**Figure 3:**
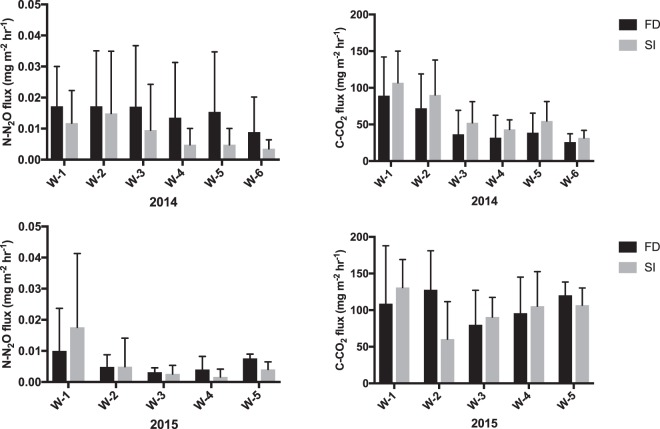
Effects of free drainage (FD) and subsurface-irrigation (SI) on N_2_O and CO_2_ fluxes. Fluxes are represented per week of effective subsurface-irrigation.

**Figure 4:**
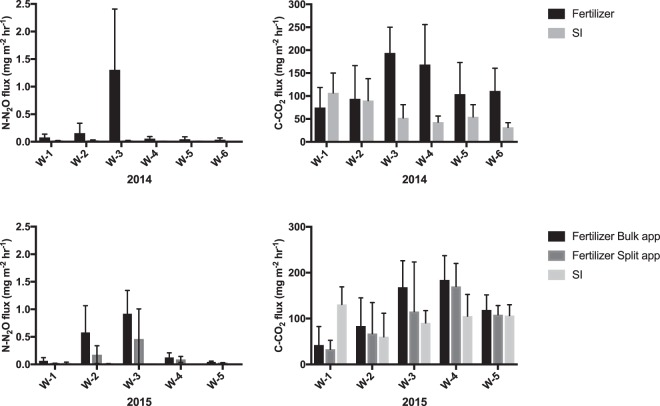
Effects of coupled fertilizer-precipitation events and of subsurface-irrigation (SI) on N_2_O and CO_2_ fluxes. Fluxes are represented per consecutive week following fertilizer application and represented per week of effective subsurface-irrigation.

Urea (CO(NH_2_); 46-0-0) was applied in all plots at the corn stage V6, when six visible leaf collars were present and occurred at the end of May-beginning of June. Increased supplies of C and inorganic N have been reported to create N_2_O pulses^31–34^. In our study, a pulsed N_2_O flux occurred within three weeks of fertilizer application and coincided with the peak CO_2_ flux in both seasons, suggesting increased N and C availability at that time (Fig. 2). Furthermore, both peak N_2_O and CO_2_ emissions occurred within 1–4 days of heavy daily rainfall events ≥24 mm. Soil aeration following saturation is important for the diffusion and release of GHGs. Continuous saturation leads to strong anaerobic conditions in the soil, which can lead to the full reduction of nitrates to N_2_, a benign gas but also can increase CH_4_ production, as observed in flooded rice fields^35^. SI systems aim to maintain a target water table depth throughout dry periods of the growing season. Results from this study suggest that prolonged anaerobic conditions in SI may in fact control gas diffusivity limiting the efflux of GHGs at the soil surface. Abalos *et al*. (ref.^36^) has previously found that increasing subsurface-irrigation frequency could mitigate GHG production by avoiding the generation of wet-dry cycles and reducing gas diffusion efficiency. Furthermore, Musarika *et al*. (ref.^37^) indicated that maintaining a water table at 30 cm below the soil surface in a radish field could reduce soil CO_2_ emissions without increasing CH_4_ production. Overall, our study indicated a relatively low impact of SI on N_2_O and CO_2_ production compared to external climatic events in a fertilized corn agro-ecosystem (Figs 3 and 4).

The substantial increase in N_2_O and CO_2_ in June was paralleled by a less pronounced increase of net CH_4_ consumption in both growing seasons (Fig. 2). At the end of June, maximum net CH_4_ consumption recorded were −0.006 and −0.01 mg C-CH_4_ m^−2^ hr^−1^, in 2014 and in 2015, for plots without split fertilizer applications. This occurred just a few weeks following fertilization, which is surprising considering previous literature indicating the suppression of methane oxidation by N-fertilizer applications^38–40^. It is possible that the increased availability of inorganic N induced a sharp increase in NH_4_-oxidizers in the soil, which led to increased consumption of CH_4_ as an alternative electron source. A previous study has indicated that methane oxidation can be mediated by NH_4_^+^-oxidizers^41^. Interestingly, in 2014, the gradual increase in net CH_4_ consumption reaching its maximum on June 16 was followed by a sharp shift from net consumption of −0.008 mg C-CH_4_ m^−2^ hr^−1^ to a net CH_4_ production of 0.004 mg C-CH_4_ m^−2^ hr^−1^, the record net CH_4_ production rate measured in that season. Considering that the peak N_2_O flux occurred the following week on June 27, it is possible that the increasing NH_4_^+^-oxidizing microbial population may have consumed available CH_4_ sources in the soil. As both methanotrophic and methanogenic processes occur simultaneously in soils^42^, the peak CH_4_ flux on June 27 may represent CH_4_ production at that time, without the counterbalancing action of methanotrophs. Interestingly, net CH_4_ production occurred in May 2014, prior to the bulk N-fertilizer applications, and in November 2014 and 2015, following harvest, which may further suggest the potential link between N_2_O producing and CH_4_ oxidizing processes in agro-ecosystems. Other studies have indeed found that cultivation and fertilizer application decreased net CH_4_ oxidation^43,44^. However, CH_4_ fluxes respective to N_2_O and CO_2_ efflux are rarely reported.

Subsurface-irrigated plots acted as net CH_4_ sinks. This contradicted our expectations of higher CH_4_ production in SI generated by the presence of anoxic soil conditions near the surface. Our findings suggest that gas diffusivity in soil pores may be a key variable in determining the final effect of water management strategies on soil GHG efflux at a field scale. Soulanges soils are primarily sandy loam within 60–180 cm of depth (Table 2). The measured saturated hydraulic conductivity (K_sat_) in the top 20 cm of soil was relatively high (3.0 × 10^−3^ cm/s), a similar value to that of sand^45^. Considering that the water table, representing the boundary between oxic and anoxic conditions was at 83 cm (SD ±11) in 2014 and 81 cm (SD ±13) in 2015, it is possible that the methane produced at the water table may have been oxidized before reaching the surface. However, this remains to be validated. Musarika *et al*. (ref.^37^) found that decreasing the water table from 50 cm to 30 cm depth from the surface reduced CH_4_ consumption possibly due to the shorter pathway for CH_4_ to reach the surface before it is oxidized. Interestingly, Van den Pol-van Dasselaar *et al*. (ref.^46^) also found the greatest CH_4_ production to occur in the upper soil horizon. Considering the higher organic matter concentrations in the top 40 cm of the soil profile in our study, raising the WT within that range is not recommended due to the higher availability of C substrates, which may lead to higher CH_4_ production. In addition, it will lead to higher water contents in the root zone, thus reducing crop yields due to excess water stress. Setting the WT at an average depth of 80 cm was suitable in our study to avoid the net production of methane and flooding the crop root zone, should heavy rains occur.

**Table 2:** Soil physical and chemical properties at depths of 0–20 cm, 20–40 cm and 40–60 cm of the experimental site.

Property	Depth		
	0–20 cm	20–40 cm	40–60 cm
**Classification**	Soulanges series; Gleysol type		
**Physical**			
Soil texture, %			
Sand	2	4	9
Silt	33	25	22
Clay	65	71	69
Bulk density, g cm^−3^	1.36	1.60	1.46
Porosity, %	49	40	45
Hydraulic conductivity K_sat_, cm × 10^−3^ s^−1^	3.00	1.55	1.70
Textural class	Sandy loam	Sandy loam	Sandy loam
**Chemical**			
Mean pH	7.0	7.2	7.3
Organic matter, %	3.51	4.51	1.32
Carbon, %	2.0	2.6	0.8
Available NO_3_-N, mg kg^−1^	5	2	1
Available NH_4_-N, mg kg^−1^	1	0	1
Mehlich III - Available P, mg kg^−1^,	98	32	9
Available K, mg kg^−1^	141	46	45
Available Al, mg kg^−1^	482	512	634
Available Ca, mg kg^−1^	1364	1120	1424
Available Mg, mg kg^−1^	157	164	374
Available Mn, mg kg^−1^	12	10	17

Chemical properties correspond to samples collected on September 8, 2015.

The peak N_2_O flux that occurred in June of each season, prior to subsurface-irrigation activation, represented ∼67% of total N_2_O fluxes in 2014 and ∼50% in 2015, whereas peak CO_2_ fluxes represented ∼10% of total CO_2_ fluxes in both seasons. This is in line with the findings of Scheer *et al*. and Liu *et al*. (refs^31,32^), who reported that peak N_2_O fluxes associated with coupled fertilizer-precipitation events accounted for as much as 50–60% of the total emissions. In accordance with previous literature^31,32^, peak fluxes in our study were associated with fertilizer application times coupled with precipitation events (Fig. 4). Our SI and FD treatment results must be considered in light of this important climatic and operational interference. The full-season analysis of our N_2_O measurements indicated significantly lower mean N_2_O flux rates in FD plots for 2014 (P < 0.01), and an inverse relationship with greater mean N_2_O flux rates in FD in 2015 (P < 0.05). However, during days of effective subsurface-irrigation, pairwise t-test mean comparisons of FD and SI treatments presented in Fig. 3 indicated significantly lower N_2_O fluxes in SI compared to FD in 2014 (P < 0.01), and no statistically significant differences in 2015. Our results support conclusions drawn by Elmi *et al*. (ref.^7^), indicating that although SI may create soil conditions favorable to increased denitrifications rates, final N_2_O efflux from the soil surface may not be increased. However, due to the substantial climatic interference on GHG fluxes, further field measurements would be highly beneficial to better elucidate SI effects on GHG fluxes.

Splitting fertilizer applications in June created a statistically significant decrease of N_2_O fluxes (P < 0.01), reducing N_2_O fluxes by 50% in our study, but did not significantly affect CO_2_ nor CH_4_ fluxes (P > 0.05) (Table 1). This may be the result of improved synchrony between corn N needs and fertilizer inputs, thereby reducing amounts of soil N available for N_2_O production. Our values are greater than those obtained in previous studies indicating a 25% N_2_O reduction from split N application^47^, a 33% N_2_O reduction^48^ and a 14% N_2_O reduction^49^ compared to regular N-fertilizer applications. Furthermore, previous split-fertilizer studies have also reported no effect of split application on N_2_O fluxes^50,51^, as well as increased N_2_O emissions^48,52^. Although our study suggests that dividing fertilizer into smaller applications may reduce N_2_O fluxes, this has to be considered in light of increased field operations and the likelihood of precipitation.

## Conclusion

Contrary to previous studies, which suggested that SI could cause an increase of gas fluxes to the atmosphere, our GHG measurements and flux calculations show that if properly managed, SI does not generate an increase in either N_2_O or CO_2_ compared to FD, and that soils within this water-table management system remain net CH_4_ sinks. Our findings suggest the importance of the water table depth when designing SI systems; increased depth of the water table from the tile drain line lengthens the diffusion pathway of GHGs to the surface, hence increasing the GHG residence time in the soil profile, and is conducive to greater CH_4_ oxidation and N_2_O reduction. Furthermore, proper timing of water table control and subsurface-irrigation respective to seasonal climatic events is crucial in determining final GHG efflux. These findings have important implications for the development of future climate change mitigation strategies, particularly as agriculture contributes over 20% of global anthropogenic GHG emissions^53^. Furthermore, our data reveal that fertilizer application timing coupled with precipitation events has the potential to trigger pulses of N_2_O fluxes accounting for up to 60% of cumulative seasonal fluxes. Our study with one growing season of data suggests that splitting dry mineral fertilizer applications may be an effective mitigation strategy, reducing N_2_O fluxes by 50%. Although considerable scientific research has focused on nitrogen losses through leaching, N losses through atmospheric efflux may be of equal importance considering the generally high global warming potential of N_2_O. A time lapse of four days between fertilizer application and intense precipitation events (24–45 mm/day) was insufficient to avoid the generation of pulses of N_2_O efflux in our study. Our assessment sets a platform for future climate change mitigation research to determine the proper dosage and timing of fertilizer applications respective to climatic events to control GHG emissions. Overall, we conclude that since crop growers install subsurface tile drainage to remove excess soil water in the spring, it is feasible to also use the subsurface drainage system as a subsurface-irrigation system, to provide supplemental water to meet crop water requirements during the dry periods of the growing season; the SI system may be a promising technology to sustain crop production and reduce NO_3_-N concentrations in drainage water, whilst avoiding the intensification of GHG fluxes.

## Methods

### Site Description and Field Management

This study was conducted over two growing seasons (2014–2015) on a 4.2 ha field of a commercial farm located in Côteau-du-Lac, Quebec, Canada (74°11′15” = lat., 45°21′0” = long.). The soil was classified as Soulanges sandy loam of the Gleysol order. It was characterized by a very fine sandy loam alluvium parent material, underlain by marine clay at depths of 60 to 180 cm. The field surface slope was approximately 0.5%. Baseline soil samples were collected in 2012. Soil physical properties including bulk density, saturated hydraulic conductivity, porosity, and textural class were measured. A second set of soil samples were collected on September 8, 2015, for which chemical analyses are presented in Table 2: organic matter content, pH, NO_3_-N, and NH_4_-N. Soil available K, Al, and P were determined using the Mehlich III method^54^. All samples were collected near each of the twelve GHG chamber locations, at depths of 0–20, 20–40 and 40–60 cm. Soil samples indicated nitrate levels (7.7 ppm (FD) and 2.9 ppm (SI)), which were within the expected range as defined by the 10-year trends for this site^6,26^. Nitrate and ammonium values by treatment and the standard deviation are included in Supplementary Fig. S2. The experimental site was under conventional tillage: chisel-plowed to 20 cm depth in the fall and harrow-disked in the spring, approximately 24 h prior to seeding. The established crop rotation for the field was one season of yellow beans, followed by three seasons of grain corn. For the two years of the study, the field was seeded with grain-corn on May 12, 2014, and on May 3, 2015, corresponding to the third and fourth seasons of the crop rotation.

### Water Table Management

Two water table management systems were studied: regular tile drainage or free drainage, and subsurface-irrigation with a target water table depth of 0.75 m. The experimental site was described by Tait *et al*. (ref.^1^). Prior to the establishment of our trial, the experimental site was set in FD for 2 years in 2012 and 2013. The site had three blocks with buffer separations of 30 m between blocks. Each block was subdivided into eight plots of 15 m by 75 m, separated by vertical plastic sheets to a depth of 1.5 m. Subsurface pipes of 0.076 m diameter were laid at the center of each plot, at an average depth of 1.0 m below the soil surface. The source of subsurface-irrigation water was groundwater obtained from a deep well. The groundwater contained no detectable nitrates, based on samples collected in 1998 (ref.^6^). The location of the well was less than 200 m from the experimental site.

In 2014, SI was implemented following a randomized complete block design (RCBD) with repeated measures. In 2015, a split-plot design with repeated measures was used to integrate a split fertilizer treatment. In FD, tile drains were left open throughout the year to facilitate the free outflow of water from the field. In SI, tile drains were left open during the spring to ensure that water drained freely from the field due to snowmelt conditions. All drains, except those in the FD plots, were then closed on June 20 in 2014 and on June 25 in 2015, using a ball-valve, once seeding and fertilizer operations were completed. The SI system was activated in July of both years, and deactivated at the end of the growing season in October of both years. Once SI was deactivated, all drains, including FD, were left open for the winter and spring. Chambers for GHG measurements were placed at approximately 3 meters from the tile drains within the 15 meter drain spacing.

### Fertilizer Applications

All decisions respective to fertilizer amounts and timing were decided by the grower, based on soil tests conducted at the site and in compliance with Quebec governmental fertilizer regulations, which are described by Rasouli *et al*. (ref.^55^). The choice of a 1-week interval between split-fertilizer applications in May-June was based on the timeframe of high N requirements for corn, demonstrated to be between V6-V8, and the time elapsed between these two stages estimated at about one week^56,57^. Providing a split application between these vegetative stages could improve the synchrony between soil N availability and corn N requirements, and limit the quantity of soil N available for N_2_O production.

Prior to the establishment of our trial, the experimental site was set in FD for 2 years in 2012 and 2013. Total N-fertilizer applications for these two years were 70 kg N ha^−1^ (2012) and 170 kg N ha^−1^ (2013). In 2014, starter fertilizer was banded at seeding at 44 kg N ha^−1^, and at 28 kg N ha^−1^ in 2015. These fertilizer amounts were calculated based on projected corn heat units (CHU) and target grain yields set at 12–14 tonnes ha^−1^. When the corn reached the vegetative growth stage with six visible leaf collars present (corn stage V6), on June 7, 2014, all plots received a second fertilizer application of 160 kg N ha^−1^. Urea (46–0–0) was broadcast and incorporated in the top 25 cm of the soil using a row crop cultivator. In 2015, for both the FD and SI treatments, half of the plots received 200 kg N ha^−1^ as one bulk application on May 29, 2015 at the V6 corn stage. The second half of the plots also received a total of 200 kg N ha^−1^. However, this amount was split evenly into two applications spaced one week apart: on May 29, 2015 and on June 3, 2015 at corn stage V8. Overall, total N-fertilizer rates were 204 kg N ha^−1^ in 2014 and 228 kg N ha^−1^ in 2015. Over the past 25 years, different fertilizer rates were tested at this site ranging from 0–270 kg N ha^−1 ^^4,6,11,26,58^. Drainage outflow, residual N, corn N uptake and yields have been measured to define the target N-fertilizer application to optimize yields whilst reducing the environmental footprint. Fertilizer rates used in this study fall within the target range, based on long-term studies conducted at the St-Emmanuel site.

### Gas sampling

Flux measurements were taken weekly during the growing season between 10 am to 4 pm. Pre-seeding and post-harvest gas samples were taken. At the identical experimental field for 2014 and 2015, two chambers were set at identical GPS-recorded locations per plot with a total of three blocks. GHG samples were taken at five time-intervals per chamber, resulting in the collection of 63 gas samples daily. Baseline GHG flux measurements were taken on November 7, 2013.

Exetainers (Labco Inc.) for GHG sampling were capped with a double septum: a Teflon/silicon septum (National Scientific, Rockwood, TN) inserted between a standard rubber septum to minimize leakage. Exetainers were evacuated in batches of ten for 60 seconds, using a single stage rotary vane mechanical vacuum pump (Welch Duoseal ® Vacuum Pump 1399, Gardner Denver Thomas Inc.).

Samples were taken using the vented non-steady state chamber method adapted from Livingston and Rochette (refs^59,60^). Each chamber cover had two openings: one with a tube fitted with a septa of 0.6 cm diameter for gas sampling and another for a vent tube of 1.6 cm of diameter. Twelve acrylic chamber frames of 0.556 m × 0.556 m × 0.140 m (W × L × H) dimensions were inserted to a depth of 10 cm in the soil, leaving 4 cm of chamber height above the surface. Frames were installed in the field after the last application of fertilizer and removed prior to harvest, so as to not disturb the sampling location during the season, and to prevent damage by field machinery. For sampling events without chamber bases, the chamber peripheries were sealed with soil to prevent the movement of air between the headspace and the atmosphere. Chamber locations were geo-referenced and remained in identical locations for all seasons. Chamber locations were set at approximately three meters from the tile drains. None of the chambers were located directly above the drains. The height of the water table was measured next to each chamber, using observation pipes located approximately 1 meter from each chamber. In tile drained soil profiles, the water table surface is flat with a narrow drop above the drain^61^. As such, due to the negligible moisture gradient within plots, chamber placement respective to drains was not expected to have a substantial impact on GHG flux measurements.

Immediately after a chamber was set onto a frame, a gas sample corresponding to time t = 0 was taken using a 20 ml syringe with a needle tip (25 gauge, 1.6 cm Benton and Dickson). Gas samples extracted from the chamber headspace were placed in evacuated 12 ml Exetainers containing 15 mg of magnesium perchlorate to absorb water vapor (Labco Inc., Wycombe, UK). Subsequent samples were taken every 15 minutes, at times t = 15, t = 30, t = 45 and t = 60 minutes. For each of these sampling times, the syringe was inserted in the chamber septa and flushed three times to homogenize the air within the headspace. At the first chamber location, three air samples outside the chamber were taken at times t = 0, t = 30 and t = 45 minutes, as controls, in addition to the five chamber samples.

### Sample measurements

Gas samples were brought back to the laboratory at McGill University for analysis through a Bruker 450-GC System (Bruker corp., Bremen, Germany). Samples were simultaneously injected onto two channels. The first was equipped with a flame ionization detector (FID) set at 300 °C, which analyzed CO_2_ and CH_4_. The second was equipped with an electron capture detector (ECD) set at 350 °C, which detected N_2_O. Helium was used as the carrier gas for the FID with a flow rate of 30 ml/min. Argon was used as the carrier gas for the ECD with a flow rate of 10 ml/min. For every 20-sample run, three gas standards were run for quality control. The analytical accuracy of the Bruker GC was as follows: ±1.1% for N_2_O, ±5.7% of CH_4_ and ±3.9% for CO_2_ with certified standards (MCRT5 mixed certified standards, Linde Canada, Mississauga, Ontario). We accepted a deviation of ±10% for our laboratory analyses, and a deviation of ≤ ±5% difference from certified standards.

The GC analysis provided raw gas flux data in parts per million (ppm). Any gas flux below the minimum threshold of 0.15 ppm, 1.7 ppm and 300 ppm for N_2_O, CH_4_ and CO_2_, respectively were excluded from the analysis (less than 1% of the 2700 measurements collected). The raw data were converted from ppm to mg of main constituent (C for CO_2_ and CH_4_, and N for N_2_O) per m^3^ of air using equation [1].

1 $${{\rm{C}}}_{{\rm{m}}}=\frac{{{\rm{C}}}_{{\rm{v}}}{\rm{MP}}}{{\rm{RT}}}$$

where C_m_ is the mass/volume concentration in mg m^−3^, C_v_ is the concentration (v/v) in ppm, M is the gram molecular weight (CO_2_ = 12 mg/mol, CH_4_ = 12 mg/mol, N_2_O = 28 mg/mol), P is the atmospheric pressure, R is the universal gas constant and T is the room temperature of the lab.

For each sampling event, the flux of each gas over the one-hour sampling time was calculated from the five concentrations taken at 15-min intervals (t = 0, 15, 30, 45 and 60 minutes), using the median flux method (MFM) as described by Mat Su (ref.^62^). The MFM technique was based on the firmly established methodologies described by Hutchinson and Mosier (ref.^63^), Parkin (ref.^64^), Rochette and Eriksen-Hamel (ref.^65^), Pedersen *et al.* (ref.^66^), Parkin *et al.* (ref.^67^), and Collier *et al.* (ref.^68^). For each two gas concentrations obtained, the slope of the linear regression was generated. As such, with five concentrations obtained over the one-hour sampling time, ten possible slopes were calculated. The median of these fluxes was taken for each chamber, using equation [2].

2 $${{\rm{f}}}_{{\rm{t}}}={\rm{H}}\,{(\frac{{\rm{\Delta }}C}{{\rm{\Delta }}t})}_{{median}}$$

where f_t_ is the GHG flux in mg/m^2^/hr, H is the chamber height, ΔC is the difference in gas concentration in mg m^−3^, and Δt is the difference of time in hours. We removed negative CO_2_ flux values, as we did not expect our soil type and conditions to produce negative CO_2_ fluxes.

### Ancillary Field and Meteorological Data

Twelve observation pipes were installed to an average depth of 1.32 m below the soil surface, approximately one meter from each of the chamber locations. On each gas sampling event, readings of the water table depth were taken using a graduated rod with a water sensor. Rainfall and air temperature data were obtained from the Côteau-du-Lac Environment Canada weather station (Station ID – 7011947) located approximately 500 m from the experimental site. Soil temperature was obtained using a hand-held thermometer of ±0.5 °C accuracy inserted in the top 10 cm of the soil (HI 98501 Checktemp ®). Soil water-filled pore space was obtained using a ThetaProbe (Model ML2x, Delta-T Devices Ltd.) inserted in the top 6 cm of the soil. The probe was calibrated to the soil of the site to achieve an average accuracy of at least ±0.02 m^3^ m^−3^. Three readings of soil water-filled pore space were recorded at the location of each GHG chamber. Averages of the three values were calculated and recorded for each gas sampling event.

### Crop Yields

Corn hybrids used in this study were Pioneer 9855 and 9411 in 2014, and Pioneer 9917 in 2015. Hybrids were chosen by the grower each year, based on projected heat units and in consultation with a seed provider. Yields per corn hybrids are not made explicitly available by the seed distributer to the growers due to guarantee liabilities and Quebec legal terms. As such, the grower did not select hybrids based on yields but rather on projected corn heat units (CHU), as a main criterion. All three hybrids used in our study (P9411, P9855 and P9917) were chosen within the 2800–2950 CHU range of the Pioneer brand products. This was considered to be a relatively narrow variation in CHU^69^. As such, the impact of CHU and of hybrid variation in this study were not expected to be significant.

In 2014, prior to combine-harvesting, crop yield samples were collected from each treatment by manually sub-sampling along three east-west lines equally spaced. Along each east-west line, corn plants were collected along a 2.5 meter length in one row. Samples were dried at 77–93 °C in a propane dryer to obtain the dry weight of the total dry biomass of stalks. Cobs were shelled and weighed to obtain the dry grain weight for each sampling location. In 2014, grain-corn yields at ∼0% moisture were 9.56 tonnes ha^−1^ (SD ±0.53) for FD, and 9.68 tonnes ha^−1^ (SD ±0.48) for SI, with a total combine-harvested yield of 10.6 tonnes ha^−1^ at the experimental site. In 2015, the grain corn was harvested by combine. Samples were weighed and adjusted to 0% moisture content. Yields in 2015 were 12.7 tonnes ha^−1^. Results of this study are consistent with long-term yields measured since 1993 at the site. Historical yield measurements for this experimental site indicated an average 4% increase of yields in SI plots compared to FD plots^5,9–11,70,71^.

### Statistical Analysis

In 2014, an RCBD was used with a total of three blocks, in which the experimental effect was the subsurface-irrigation treatment. The repeated statement was sampling day with a total of 21 measurements over the season. GHG measurements were taken at five time-intervals (t = 0, 15, 30, 45 and 60 minutes) from two chambers per block replicated over time. Measurements were taken on a weekly basis from May 15 to October 13. A post-harvest sample was taken on November 4, 2014.

In 2015, the plots were further subdivided into subplots which either received one bulk fertilizer application or a split fertilizer application. GHG measurements were taken at the same GPS-referenced locations in the plots as in 2014, at five time-intervals from one chamber per subplot per block, with a total of 3 blocks. Our repeated statement was sampling day with a total of 24 time points during the season from April 27 to November 3, 2015, with weekly measurements.

The two seasonal datasets were analyzed individually. The JMP Statistical Visualization Software (JMP 11.2.0) was used to perform the analysis of variance (ANOVA). The assumption of normal distribution of residual errors was assessed by the Wilk-Shapiro test. Nitrous oxide data were log10(x + 10)-transformed to meet the assumptions of a normal distribution. Homogeneity of variance was assessed using Levene’s test. Negative CO_2_ flux values were removed, and this was no more than 2.5% of the dataset. In 2014, sampling day and subsurface-irrigation were defined as fixed variables, and block was specified as a random variable. In 2015, sampling day, subsurface-irrigation and fertilizer were indicated as fixed variables and block was included as a random variable. Statistically insignificant interactions amongst variables were removed from the model. All models were significant at *P* < 0.05. ANOVA results are presented in Supplementary Table S1.

Separate pairwise t-tests were conducted on sampling days during effective subsurface-irrigation; subsurface-irrigation was continuous from the end of July to October in 2014 and from July to October in 2015. During effective subsurface-irrigation, gas samples were collected on six sampling days in 2014 and on five sampling days in 2015.

Pearson’s correlations were performed to determine the relationship between soil temperature (°C) and WFPS (%) with GHG fluxes. Linear regressions were performed with GHG flux rates as the main factor. Results are presented in Supplementary Table S2 and Fig. S1.

## Supplementary information

Supplementary Information

## Data Availability

The authors declare that data supporting the findings of this study are available within this article and its Supplementary Information, and all additional data are available from the corresponding author on reasonable request.
