# Supplementary material for: Water table management and fertilizer application impacts on CO2, N2O and CH4 fluxes in a corn agro-ecosystem
Source: Sci Rep. 2019 Feb 25;9:2692. doi: 10.1038/s41598-019-39046-z (PMC6389930; doi:10.1038/s41598-019-39046-z)

Supplementary Information

Water table management and fertilizer application impacts on CO_2, N_2O and CH_4 fluxes in a corn agro-ecosystem

Cynthia M. Crézé and Chandra A. Madramootoo

ANOVA Models by year

Year 1

Fixed variables
day
subsurface-irrigation
day*subsurface-irrigation

Random variables
block
day*block
block*subsurface-irrigation
day*block*subsurface-irrigation = residual error

Year 2

Fixed variables
day
subsurface-irrigation
fertilizer
day*subsurface-irrigation
day*fertilizer
subsurface-irrigation*fertilizer

Random variables
block
day*block
block*subsurface-irrigation
block*fertilizer
day*block*subsurface-irrigation
day*block*fertilizer
block*subsurface-irrigation*fertilizer
day*block*subsurface-irrigation*fertilizer=residual error

Supplementary Table S1 **Fixed effects test results of N_2O, CO_2 and CH_4 flux data using the ANOVA procedure of JMP-SAS. Homogeneity of variance was assessed using Levene's test. The assumption of normal distribution of residual errors was assessed by the Wilk-Shapiro test. Nitrous oxide data was log10(x+10)-transformed to meet the assumptions. Statistically insignificant interactions amongst variables were removed from the model.**

		N_2O			CO_2			CH_4		
		DF	F ratio	P > F	DF	F ratio	P > F	DF	F ratio	P > F
**2014**	**Day**	20	49.0355	**<0.0001*** **	20	18.4993	**<0.0001*** **	20	3.1880	**<0.0001*** **
	**Subsurface-irrigation (SI)**	1	15.6295	**0.0001*** **	1	6.5607	**0.0111** **	1	0.5021	0.4793
	**D*SI**	20	20.6771	**<0.0001*** **		N.S.S.			N.S.S.	
**2015**	**Day**	23	23.6604	**<0.0001*** **	23	20.2544	**<0.0001*** **	23	1.2719	0.1863
	**Subsurface-irrigation (SI)**	1	5.8979	**0.0160** **	1	14.9886	**0.0001*** **	1	4.2877	**0.0394** **
	**Fertilizer**	1	10.4547	**0.0014*** **	1	0.0168	0.8969	1	0.3359	0.5627
	**D*SI**	23	3.1633	**<0.0001*** **	23	2.3519	**0.0007*** **		N.S.S.	
	**D*F**	23	3.5507	**<0.0001*** **		N.S.S.			N.S.S.	
	**SI*F**		N.S.S.		1	6.0299	**0.0148** **		N.S.S.	
	**D*SI*F**		N.S.S.			N.S.S.			N.S.S.	

**p<0.05-0.01; ***p<0.01; N.S.S. = not statistically significant

Supplementary Table S2 **Pearson's correlations between mean daily N_2O, CO_2 and CH_4 and soil temperature (°C) and water filled pore space (WFPS in %) for 2014 and 2015. n = 45.**

	Soil WFPS (%)		Soil temperature (°C)	
	r	p	r	p
**N_2O flux** (mg N-N_2O.m^{-2}.hr^{-1})	0.2802	0.0623	**0.52*** **	0.0003
**CO_2 flux** (mg C-CO_2.m^{-2}.hr^{-1})	0.02192	0.8864	**0.7415*** **	<0.0001
**CH_4 flux** (mg C-CH_4.m^{-2}.hr^{-1})	-0.03492	0.8199	-0.2356	0.1192

**p<0.05-0.01; ***p<0.01

Supplementary Table S3 **Mean monthly precipitation and air temperature during the growing seasons of 2014 and 2015, compared to the long-term (40-year) mean measured at the Côteau-du-Lac weather station.**

**Month**	**Mean monthly air temperature (°C)**		
	2014	2015	40-Year Average
May	13.8	16.3	13.1
June	19.4	17.2	18.0
July	19.9	20.4	20.4
Aug	18.9	19.9	19.2
Sep	15.0	18.1	14.6
**Mean**	**17.4**	**18.4**	**17.1**

**Month**	**Precipitation amount (mm)**		
	2014	2015	40-Year Average
May	85.3	56.8	86.5
Jun	132.0	135.1	98.7
Jul	61.9	87.9	101.0
Aug	51.4	74.5	93.4
Sep	42.6	75.3	94.8
**Total**	**464.9**	**494.0**	**547.1**

Supplementary Figure S1 **Linear regression equations between mean daily GHG fluxes and mean daily soil temperature (°C) and water filled pore space (WFPS in %) for 2014 and 2015.**

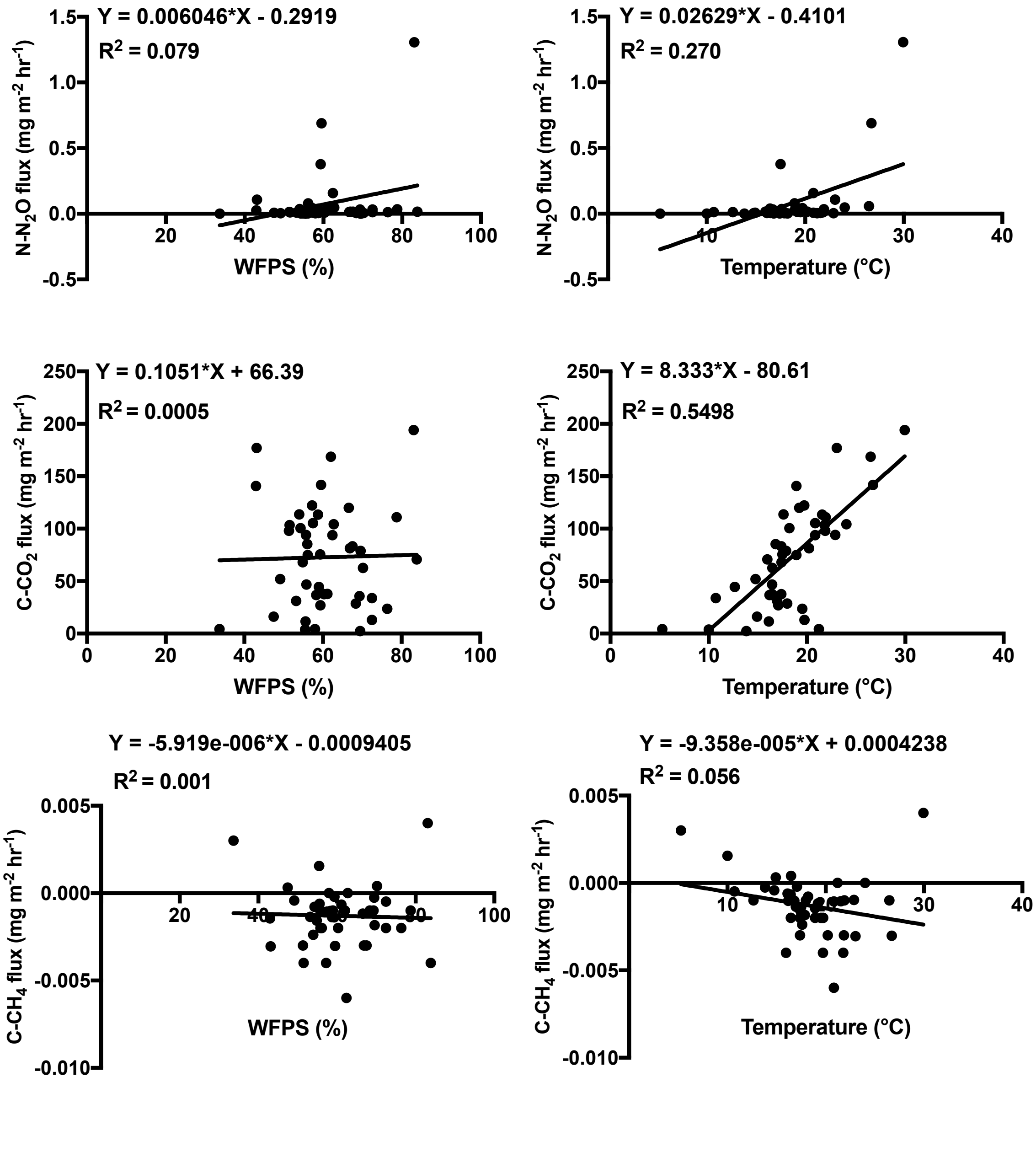

Supplementary Figure S2 **Season 2015 average soil nitrate (a) and ammonium (b) at depth 0-20 cm, 20-40 cm and 40-60 cm, and standard deviation by water treatment**

(a)

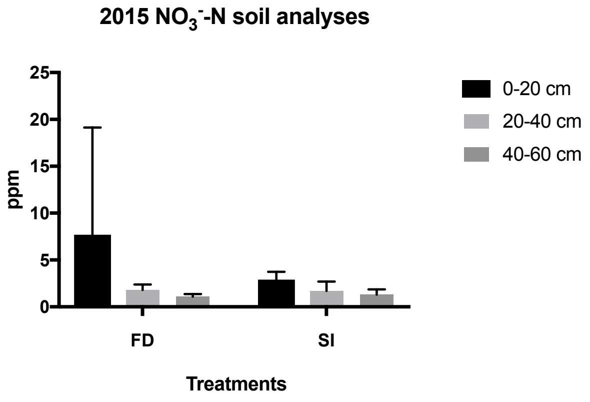

(b)

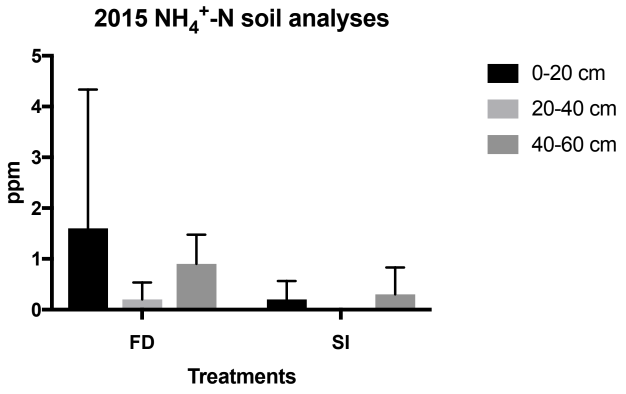

Supplement: Supplementary file 1 — Supplementary Information [file 41598_2019_39046_MOESM1_ESM.pdf]
